# Oral Health as Entry to Improve Population Health—Research Implications

**DOI:** 10.3390/healthcare14132015

**Published:** 2026-07-06

**Authors:** Fred S. Ferguson, Mark E. Moss

**Affiliations:** 1 Health Migration Consulting, Inc., East Setauket, NY 11733, USA; ferguson@healthmigration.com; 2 School of Dental Medicine, East Carolina University, Greenville, NC 27834, USA

**Keywords:** oral health, population health, social determinants of health, preventive care, medical–dental integration, predictive modeling, health equity, consumer engagement

## Abstract

**Highlights:**

**What is the main finding?**

- A research framework to address six key pillars is presented to link oral health with improved systemic health.

**What are the implications of the main finding?**

- Using innovation and technology, patient-reported behaviors linked with clinician-observed oral health status may enable earlier identification of risk, more timely feedback, and improved engagement.
- This can translate to improved health outcomes that go beyond oral health.

**Abstract:**

Escalating healthcare costs and persistent disparities in outcomes have been linked to delayed engagement in preventive care and the ongoing influence of social determinants of health (SDoH). Current care models often assume that individuals can effectively manage their health and seek care at appropriate times, despite evidence that contextual constraints limit this capacity. This perspective proposes that oral health may serve as a practical and scalable entry point for earlier engagement in preventive care. Oral health problems are highly prevalent, behaviorally influenced, clinically observable, and associated with systemic health conditions. We present a six-pillar conceptual framework linking consumer-reported behaviors with clinician-observed oral health status to support risk identification, engagement, and population health strategies. The framework integrates behavioral science, predictive modeling, social risk factors, digital health infrastructure, and policy innovation. We outline a research agenda, identify implementation challenges, and discuss equity considerations. While oral–systemic relationships are well-documented as associations, further prospective and interventional studies are needed to establish causality and population-level impact.

## 1. Introduction

Healthcare systems continue to face increasing costs, suboptimal outcomes, and persistent inequities. A key contributor is delayed engagement in preventive care, particularly among populations affected by social, economic, and environmental constraints.

Evidence consistently demonstrates that individual behaviors, daily habits, and environmental conditions significantly influence health outcomes [1]. However, many care systems remain oriented toward reactive disease management rather than proactive prevention. Whole-person health emphasizes the integration of biological, behavioral, and social influences [2]. Yet there remains a lack of structured processes to support individuals in sustained health engagement outside clinical encounters.

In this manuscript, we use the term “consumer” to emphasize the role of individuals as active participants in health-related decision-making in daily life. We recognize that this term has limitations, as it may imply autonomy that is constrained by structural factors such as poverty, limited access to care, disability, or health literacy barriers. Accordingly, the term is used to highlight engagement while acknowledging inequities in opportunity and access.

We propose that oral health offers a practical entry point for addressing gaps in preventive engagement. This paper introduces a six-pillar conceptual framework and outlines a research agenda to advance oral health as part of a whole-person, preventive health model of care.

## 2. Why Oral Health as an Entry Point for Prevention

Oral health is uniquely suited as an early engagement domain. Behaviors influence clinically observable early manifestations of oral disease across the lifespan [3]. Changes in the oral microbiome can lead to periodontitis [4] and dental caries [5]. These conditions have been linked to systemic health [6]. Oral health also intersects with social determinants such as income, education, access to care, and geographic location [7]. These characteristics make it a practical domain for early detection of risk and structured consumer engagement.

It is important to note that oral–systemic relationships are largely associative and often bidirectional, influenced by shared risk factors such as diet, smoking, inflammation, and socioeconomic conditions. While these associations are well established, causal pathways require further investigation through longitudinal and interventional studies.

## 3. Conceptual Approach and Framework Development

This framework was developed through an integrative synthesis of multiple domains, including behavioral science and health behavior theory; population health and social determinants of health frameworks; the medical–dental integration literature; predictive analytics and digital health innovation; and value-based care models. Across these diverse fields, six recurring themes emerged as foundational pillars: the central role of behavior in shaping health outcomes; the predictive value of integrated, cross-sector data; the profound influence of social determinants; the importance of meaningful consumer–clinician collaboration; the enabling role of robust digital infrastructure; and the necessity of aligned policy and payment models to support sustainable change. While this perspective does not constitute a formal systematic review, it offers a conceptually grounded framework intended to inform and guide future research, policy development, and implementation efforts.

## 4. A Six-Pillar Framework for Advancing Oral Health and Population Health

### Pillar 1: Consumer Engagement and Pre-Clinical Behavior Science

This pillar emphasizes understanding behaviors before disease onset and supporting sustained engagement in preventive practices. By leveraging behavioral science principles, targeted education, and personalized engagement strategies, it emphasizes research on ways to foster adoption of proactive self-management habits such as toothbrushing, healthy diet and snacking, and regular visits to a health professional. Strategic research gaps include the role of lifelong oral health habits on other health habits and conditions such as smoking and obesity.

### Pillar 2: Oral–Systemic Predictive Modeling

This pillar explores whether oral health indicators, alone or combined with systemic data, can improve risk prediction. Candidate variables include markers of periodontal health, dental caries, tooth loss, xerostomia and salivary flow, oral microbiome measures and oral-health-related quality of life. These data may support earlier identification of individuals at risk for systemic disease, though causal implications require further validation.

### Pillar 3: Social Determinants as Drivers of Risk

This pillar addresses how social and environmental conditions shape oral health outcomes and engagement. Key domains include income and insurance status; education and health literacy; geographic access to care; food environment; social support and stress. Research is needed to tailor interventions specific to the social and environmental contexts that can limit opportunities for healthy decision-making.

### Pillar 4: Two-Step Consumer–Clinician Data Model

This pillar proposes a structured, bidirectional model of data integration.

Step 1: Consumer-reported data collected through digital tools (e.g., mobile applications, web platforms) on behaviors, symptoms, and preferences.

Step 2: Periodic clinician validation through examination and structured assessment.

Research can test the effectiveness of this two-step approach, providing a feedback loop, that may result in enhanced engagement and adherence leading to improved outcomes based on risk tracking and early detection of disease.

### Pillar 5: Technology and Interoperability

This pillar focuses on building the digital infrastructure and data standards needed to connect systems, providers, and patients. Interoperable platforms can provide seamless sharing of health information across medical and dental environments while maintaining security and privacy. The creation of scalable, connected ecosystems are the basis for research to support innovation, improve efficiency, and enable data-driven decision-making across the oral health landscape.

### Pillar 6: Policy and Payment Innovation

Preventive models require alignment with policy and reimbursement structures. This includes value-based payment models that provide incentives for early intervention. Research is needed to demonstrate financial sustainability as well as evaluation of factors that contribute to a system-wide adoption of preventive practices, ultimately supporting a more effective and equitable oral health system.

## 5. Research Agenda and Expanded Framework

Opportunities for research and innovation are emerging across the landscape of oral healthcare and the wider health system. Research rigor must assure the reliability of self-reported data, calibration of clinical measures and accuracy of clinically meaningful outcomes using standardized protocols. By integrating technological advancements, addressing social determinants, and fostering consumer engagement, dentistry can play a central role in transforming healthcare systems. Oral health’s accessibility, behavioral transparency, and clinical measurability make it an ideal starting point for predictive and preventive care models. The combination of digital tools, interdisciplinary collaboration, and innovative care delivery frameworks has the potential to significantly improve outcomes while reducing costs. Table 1 identifies some ways the six-pillar framework can be applied.

The pillars align with active trends in healthcare adoption of digital technology [8]. For example, the use of “big data” models has been proposed to address preventable hospitalizations [9]. The integration of oral health tracking into general healthcare has been promoted to prevent potential linkages such as diabetes-related complications [10,11]. Such approaches may help test whether oral health behaviors, clinical oral health measures, and integrated monitoring contribute to earlier risk identification and improved systemic health outcomes. From a research perspective, oral health behaviors are relatively easy to assess. The framework offers some starting points. Future studies are needed to examine contextual factors that contribute to improved oral health. These studies can examine impacts across the lifespan from infancy to old age.

Beginning in early childhood, oral health self-management quality can be easily assessed from consumers and then confirmed via dental examination. Pending prospective validation, this may support risk stratification that can lead to improved systemic health and quality of life. The approach embraces a strategy of structured engagement between consumers and healthcare providers for prevention that could have far-reaching benefits.

## 6. Implementation Challenges and Equity Considerations

Research can address implementation challenges [12]. Clinical workload issues [13] and the potential for algorithmic bias [14] can result with the adoption of new technology. Likewise, health equity could worsen if efforts are not explicitly addressing the barriers that marginalized populations face with respect to accessing technology and resources. Efforts are needed to assure equitable implementation. For example, community health worker support or other non-digital alternatives may be required. Accessibility and privacy safeguard issues may need tailored solutions for older adults or those with disabilities.

Oral health is relevant across the life course and across socioeconomic groups, although opportunities for prevention and care are strongly shaped by income, education, geography, disability, and insurance coverage [15]. From infancy, common childcare behaviors, habits, and lifestyle choices have an impact on the oral microbiome and this may play an important role in an individual’s health journey [3,16]. With aging and the impact of social and environmental factors [17], these influences may evolve to become more significantly linked to common chronic illnesses such as diabetes, cardiovascular disease, high blood pressure, adverse pregnancy outcomes, and cognitive health concerns [18]. Harmful lifestyle choices such as tobacco, recreational drug use and alcohol abuse are significant cofactors connecting oral and systemic health [19]. Research is needed to untangle the complexity of these social–bio–behavioral connections.

Theory-based, conceptually driven approaches may help guide our understanding. For example, healthy decision-making has been framed in terms of Capability, Opportunity, and Motivation [20]. Social and environmental constraints can limit opportunities for health [21]. As external constraints, social determinants of health (SDoH) can be regarded as “Social Drivers of Risk.” This perspective enables appreciation of consumer-related influences and associated clinically identifiable health outcomes as a strategy to better engage consumers to advance healthcare by integrating these linkages into a predictive healthcare model.

## 7. Policy and Payment Implications

Economic return on investment represents another challenge. Payors (employers, state government, commercial) are in the best position to engage consumers and oral healthcare practitioners via web-based technology to initiate an effective partnership between consumers and providers. Web-based tools and mobile-first designs can collect real-time data to drive predictive interventions and enhance equity among underserved communities. System dynamics modeling can build simulations that result in actionable insights [22]. US healthcare systems have shown improved efficiencies and cost reduction in chronic care management by co-location of medical and dental services and gathering and sharing patient data [23]. However, in many settings, co-location remains in the reactive, disease-driven mode and the potential preventive impact remains unrealized due to the lack of “forward looking” data that may be provided through a simple oral health assessment.

## 8. Conclusions

Testable hypotheses can arise from the proposed six-pillar framework. Beginning with improved shared engagement and a simple, two-factor consumer-led oral health data process, healthcare can engage consumers earlier and more equitably. This engagement supports the alignment of innovation with meaningful care outcomes. Data tools can provide real-time, relevant data to support healthy behaviors and initiative-taking. These data tools can be tailored to bridge the gap between lived experiences (SDoH) and clinical care.

Oral health provides a starting point for structured consumer engagement to be prioritized, incentivized, and seamlessly integrated into health systems to shift from a reactive to a more effective predictive healthcare model. Active research is needed to enhance the innovations that show promise for integrating oral health as part of a whole-person model of health. Improved outcomes and reduced costs may result if these models are validated and implemented equitably. This approach to oral health offers a foundation for research that may enable a paradigm shift toward a system of care that leverages innovative use of digital tools and fosters shared accountability among consumers, providers, policy makers and payors.

## Figures and Tables

**Table 1:** Key research questions to drive innovation in healthcare.

Pillar	Key Research Questions	Study Design	Expected Outputs	Examples
**1. Consumer Engagement & Pre-Clinical Behavior Science** Engage individuals before disease onset	What drives sustained engagement? How early can signals be detected?	Longitudinal cohort or Pragmatic trial to assess self-management and behavior change	Validated behavioral metrics Early intervention frameworks Increased preventive engagement	Longitudinal study of text messaging via mHealth tool to improve toothbrushing habits to reduce plaque score in adolescents.
**2. Oral–Systemic** **Predictive Modeling** Establish oral health as predictor of chronic disease	Which oral findings predict disease? Does combined data improve prediction?	Longitudinal cohort Predictive analytics Causal modeling of markers of oral disease and chronic disease	Predictive risk tools Earlier diagnosis of chronic disease Improved outcomes and cost reduction	Longitudinal study of periodontal disease, markers of inflammation, and onset of dementia.
**3. SDoH as Social Drivers of Risk** Make SDoH measurable and actionable	How do SDoH manifest in oral health? What indicators reflect social risk?	Community-based participatory methods with a focus on underserved and marginalized populations	Quantifiable SDoH Indicators Equity-focused care models Reduced disparities	Mixed methods approach to reduce sugar sweetened beverage intake in vulnerable and underserved communities.
**4. Two-Step Consumer–Clinician Data Model** Operationalize partnership care model	Does combining self-report + clinical validation improve outcomes? What is optimal validation frequency?	Randomized clinical trials or Pragmatic trials in clinical populations	Evidence-based engagement model Improved prevention and early detection Reduced reactive care	Clinical trial showing improved toothbrushing, gingival bleeding scores, and serum markers of inflammation.
**5. Technology & ** **Interoperability** Enable real-time data-driven care	What tools support continuous tracking? What protocols best ensure secure data sharing with minimal disruption to clinical care?	Implementation studies System level analytics Assess adoption rates	Interoperable health systems Real-time predictive analytics Improved care coordination	Adoption of machine learning to link periodontal measures of bone loss with HbA1c and diabetes onset.
**6. Policy & Payment** **Innovation** Align incentives with prevention	What payment models support prevention? How to incentivize engagement?	Policy evaluation Health system metrics of cost effectiveness, return on investment	Policy-relevant evidence Incentivized preventive care models Lower healthcare costs	Use of a social worker to improve medical–dental efficiencies through care coordination.

SDoH—Social Determinants of Health.

## Data Availability

No new data were created or analyzed in this study.

## Abbreviations

The following abbreviations are used in this manuscript:

SDoH	Social Determinants of Health
